# A Comprehensive Analysis of Genomics and Metagenomics in a Heterozygote Familial Hypercholesterolemia Family

**DOI:** 10.3389/fcimb.2021.605954

**Published:** 2021-03-02

**Authors:** Honghong Liu, Ye Jin, Ran Tian, Siqin Feng, Shuyang Zhang, Chenhong Zhang

**Affiliations:** ^1^ Department of Cardiology, Peking Union Medical College Hospital, Peking Union Medical College & Chinese Academy of Medical Sciences, Beijing, China; ^2^ State Key Laboratory of Microbial Metabolism, School of Life Sciences and Biotechnology, Shanghai Jiao Tong University, Shanghai, China

**Keywords:** familial hypercholesterolemia, *LDLR* gene mutation, whole exome sequencing, gut microbiota, *Prevotella dentalis*

## Abstract

Familial hypercholesterolemia (FH) is an inherited rare disease leading to markedly elevated low-density lipoprotein cholesterol (LDL-C) levels and increased risk for cardiovascular event. Gut microbiota has been implicated as a pivotal contributing factor in hyperlipidemia, however, its role in FH remains elusive. We performed whole-exome and metagenomics sequencing on a family with 22 members in which myocardial infarctions occurred at a young age with unclear etiology. We confirmed the missense mutation of *LDLR* c.1723C>T accounted for the abnormal cholesterol metabolism in the family through co-segregation analysis. In addition, *Prevotella dentalis* was found elevated and strongly associated with LDL-C level in FH family members with mutation of *LDLR* c.1723C>T compared to unaffected members with hyperlipidemia. Overall, our work suggests that whole-exome sequencing can facilitate identification of disease-causing variants and enable preventive treatment of FH. Our metagenomics analysis provides early insights into potential contributions of host-microbe interactions in genetic and common hypercholesterolemia.

## Introduction

Familial hypercholesterolemia (MIM#143890), an inherited rare disease mainly caused by the mutation of low-density lipoprotein receptor (*LDLR*) gene or apolipoprotein B (*APOB*) gene in autosomal dominant pattern, which is typically associated with premature coronary artery disease (CAD) (Defesche et al., 2017). Based on the presence of FH-causing alleles, the prevalence of the disease has been estimated worldwide to be 1/1,000,000 for homozygotes (Santos et al., 2016). The risk for the development of CAD in patients with untreated heterozygous familial hypercholesterolemia (HeFH) is 20 times higher than that in treated patients (Khera et al., 2016). Although *PCSK9* inhibitors such as evolocumab emerged as a new LDL-C lowering therapy and showed significant reductions in cardiovascular outcomes (Sabatine et al., 2015), FH remains underdiagnosed and undertreated in most countries, which need further effort to better manage (Nordestgaard et al., 2013). In recent years, emerged evidence shows that the gut microbiota, as an internalized environmental factor, plays an essential role in human cardiovascular disease (Brown and Hazen, 2018). Moreover, compelling evidence from animal and human studies suggests that genotype also affects the composition of gut microbiome (Goodrich et al., 2014). Thus, the effect and interaction of the host genotype and gut microbiota in lipid metabolic phenotypes remain elusive, which might provide important information for treatment of hyperlipidemia and CAD.

Here, we reported on an extended myocardial infarction (MI) family, which remained without diagnosis with FH until whole-exome sequencing (WES) was performed. We identified the pathogenic *LDLR* variant accounting for the abnormally elevated LDL-C level. In particular, we investigated metagenomic profile of this family by comparing FH members with unaffected members to explore the role of gut microbiota in genetically predisposed hypercholesterolemia. The current study illustrates the need for a more systematic employment of multi-omics analysis of FH to increase the awareness and timely initiation of medical treatment.

## Materials and Methods

### Study Subjects

We recruited a three-generation Chinese family with FH from the Peking Union Medical College Hospital in 2019. All subjects analyzed in this study gave written informed consent before participating. Venous fasting blood was drawn in the morning the day after recruitment. Then, the laboratory data including lipid profile (total cholesterol, low-density lipoprotein cholesterol, high-density lipoprotein cholesterol, and triglycerides) were measured immediately. Stool samples freshly collected from each participant under instruction were immediately transported to the laboratory and frozen at −80°C immediately. The local Ethical Committee (JS-1233, Peking Union Medical College Hospital) approved the study.

### Whole-Exome Sequencing Analysis and Mutation Screening

Genomic DNA was extracted with QIAamp DNA Blood Mini kit (Qiagen, Hilden, Germany) following the manufacturer’s protocol. The extracted DNA was fragmented randomly and then purified using magnetic particle method. DNA fragments were ligated with adaptors and captured by probes of the IDT XGen Exome Research Panel (IDT, Lowa, USA) targeting about 19,396 genes. The DNA libraries after enrichment and purification were sequenced on the NovaSeq 6000 sequencer according to the manufacturer’s instructions (Illumina, San Diego, USA).

All reads were aligned to the reference human genome (UCSC hg19) by Burrows-Wheeler Aligner (BWA, v.0.5.9-r16) (Li and Durbin, 2010). Picard (v2.5.0) was used to mark duplicate and low‐quality reads. Genome Analysis Toolkit (GATK, v4.0.6.0) was used for variant calling (McKenna et al., 2010). Resulting variants were annotated with Annovar (2018Apr16) to evaluate their effect on coding sequences, allele frequency in the general population, and the predicted level of pathogenicity (Wang et al., 2010). Allele frequencies were reported using the GnomAD, TOPMED, ExAC, and G1000. Minor allele frequencies, prior evidence of disease causality, and pathogenicity scores predicted by SIFT (Ng and Henikoff, 2003) and Polyphen‐2 (Adzhubei et al., 2013) were used to prioritize variants during the curation of the filtered variant list. We put emphasis on variants in genes with strong clinical evidence. The chosen variants were then verified using Sanger sequencing.

### Metagenomics Sequencing and Analysis

DNA extraction was performed using the Qiagen QIAamp DNA Stool Mini Kit (Qiagen) according to the manufacturer’s instructions. DNA library construction was performed following the manufacturer’s instruction (Illumina). All samples were sequenced based on the Illumina HiSeq X Ten platform. High-quality paired-end reads from each sample were used for *de novo* assembly with IDBA_UD v1.1.0 (Peng et al., 2012) into contigs longer than 500 bp. The high-quality reads from each sample were aligned against the gene catalogue by SOAP2 v2.22 (Li et al., 2009) using the criterion of “identity > 90%.” Prediction of genes from each sample was processed based on Meta-GeneMark v.2.1 (Zhu et al., 2010). A non-redundant gene catalog of 1,048,575 microbial genes was constructed with CD-HIT v4.6.4. The high-quality reads distribution of different samples was aligned with Bowtie2 (Langmead and Salzberg, 2012).

Putative sequences were translated from the gene catalog and aligned against the KEGG databases (release 89.1). MEGAN v4.6 (Afshinnekoo et al., 2015) was used for taxonomy annotation. Redundancy analyses was computed on transformed bray-curtis distances, the significance was assessed by permutational multivariate analysis of variance (PerMANOVA, 999 permutations) in R (3.6.0). All differential abundances of genes, species and KO screening were tested using the Wilcoxon rank sum test. Wherever mentioned, the Benjamini-Hochberg method was used to control the FDR.

## Results

### Clinical Case Description

**Figure 1:**
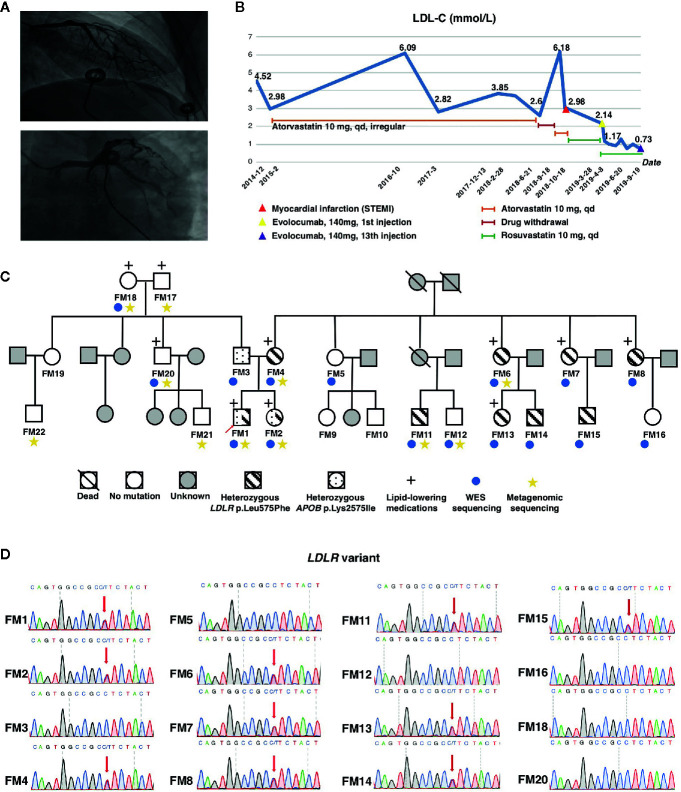
Pedigree of the HeFH family and mutation screening. **(A)** Coronary artery angiography showing obstruction in the left anterior descending artery (LAD) and left circumflex artery (LCX) of the proband. **(B)** LDL-C changes of the proband during recent 5 years. **(C)** Pedigree of the family members and their corresponding phenotypes. **(D)** Sequencing results of the *LDLR c.1723C>T* variant. Red arrow indicates heterozygous mutations. LDL-C, low-density lipoprotein cholesterol.

The proband was a 33-years-old male (FM 1), who was diagnosed with acute ST-elevated MI 5 months ago. Emergency coronary angiography showed that 60 to 80% obstruction of left anterior descending artery (LAD) and 50% stenosis of left circumflex artery (LCX) (**Figure 1A**). The patient was presented to our hospital due to chest pain and fatigue during physical exercise recurrently. Electrocardiogram and laboratory cardiac enzyme test were all normal. The myocardial perfusion tomographic imaging (adenosine) revealed transverse, coronal, and sagittal planes showing areas of reduced radioactivity distribution in the basement of the posterior lateral wall of the left ventricle. His hyperlipidemia was first diagnosed at age 28, he had taken atorvastatin (10 mg, q.d.) irregularly since then for 4 years, and the LDL-C level ranged between 2.6 to 3.8 mmol/L. Unexpectedly, his LDL-C level rose to 6.18 mmol/L 1 month before MI owing to drug withdrawal (**Figure 1B**).

His mother experienced lethal MI at the age 50. She was also diagnosed with type 2 diabetes mellitus (T2DM) and hypertension. His several maternal aunties had hyperlipidemia and one of them was died of MI at the age 41. His father had T2DM at age 50. His sister was diagnosed of hypertension, T2DM, and hyperlipidemia at age of 29.

### Identification of Heterozygote Low-Density Lipoprotein Receptor Mutant in this Familial Hypercholesterolemia Family

Since the patient had a family history of early-onset coronary heart disease, we highly suspect that his dyslipidemia was genetically related. We recruited the remaining 21 family members to investigate the genetic mechanism. The basic demographic data were summarized in **Table 1**. We performed WES analysis on this proband and other 15 family members (**Figure 1C**). We found the proband carried two deleterious mutations including *LDLR* (c.1723C>T, p.Leu575Phe) and *APOB* (c.7724A>T, p.Lys2575Ile) (**Table 2**). We identified that pattern of inheritance of heterozygous *LDLR* variants was fully co-segregated with the elevated levels of LDL‐C in this family and the causality was further verified by Sanger sequencing. The *APOB* c.7724A>T variant carried by the proband was inherited from the father who had normal cholesterol level. Meanwhile, the *APOB* mutation was not identified in FM18 and FM 20 who had elevated LDL-C level. Therefore, according to the principle of co-segregation, we exclude the pathogenicity of *APOB* mutation (**Figures 1C, D**). Finally, the proband and other nine family members were diagnosed with heterozygote familial hypercholesterolemia according DLCN (Dutch Lipid Clinic Network) criteria after genomic screening (Umans-Eckenhausen et al., 2001). After being diagnosed with FH, the proband received evolocumab treatment (140 mg) for 5 months and the LDL-C level dropped from 2.14 to 0.73 mmol/L immediately (**Figure 1B**).

**Table 1:** Clinical characteristics of enrolled FH subjects.

	Age	Sex	BMI kg/m^2^	LDL-C <3.37 mmol/L	TC (2.85–5.7) mmol/L	HDL-C (0.93–1.81) mmol/L	TG (0.45–1.7) mmol/L	Medical History	Lipid Lowering Drug	Diagnosis
FM2	32	F	25.8	2.35	3.99	1.26	1.59	CAD, HTN	Rosuvastatin, 10 mg	FH
FM3	54	M	24.9	2.93	4.84	1.26	1.01	T2DM	–	**-**
FM4	57	F	24.8	2.90	5.17	1.19	1.16	MI, HTN, T2DM	Rosuvastatin, 10 mg	FH
FM5	55	F	27.3	2.57	4.35	1.38	0.96	HTN	–	**-**
FM6	49	F	22.1	3.28	5.45	1.55	1.02	HLP	Simvastatin, 20 mg	FH
FM7	48	F	19.9	2.83	5.36	1.99	0.45	HTN, HLP	Rosuvastatin, 10 mg	FH
FM8	45	F	19.6	3.15	5.26	1.66	0.85	HLP	Rosuvastatin, 10 mg Ezetimibe, 10 mg	FH
FM9	32	F	22	2.57	4.08	0.99	1.14	**-**	–	–
FM10	28	M	27.2	3.28	4.86	0.90	1.61	**-**	–	–
FM11	28	M	28	5.33	7	1.04	0.88	**-**	–	FH
FM12	25	M	25.3	3.64	5.82	1.65	0.89	HTN	–	HLP
FM13	26	F	23.7	2.76	4.96	1.74	0.59	HLP	Rosuvastatin, 10 mg Ezetimibe, 10 mg	FH
FM14	20	M	26.5	4.12	5.74	1.38	0.66	–	–	FH
FM15	21	M	22.5	4.69	5.86	1.04	0.5	–	–	FH
FM16	24	F	20.2	2.28	4.78	2.03	0.8	–	–	–
FM17	82	M	29.4	3.62	6.19	1.03	3.3	HTN, T2DM, HLP	Rosuvastatin, 10 mg	HLP
FM18	80	F	24.7	4.29	6.28	1.35	1.2	HLP	Rosuvastatin, 10 mg	HLP
FM19	48	F	31.3	2.89	4.56	0.99	1.61	–	–	–
FM20	54	M	27.7	3.55	5.65	1.18	2.05	HTN, HLP	Rosuvastatin, 10 mg	HLP
FM21	28	M	34.3	3.63	5.23	1.08	1.46	–	–	HLP
FM22	22	M	28.4	3.90	5.50	1.07	1.08	–	–	HLP

F, female; M, male; LDL-C, low-density lipoprotein cholesterol, mmol/L; TC, total cholesterol, mmol/L; HDL-C, high-density lipoprotein cholesterol, mmol/L; TG, triglyceride, mmol/L; HLP, hyperlipidemia; FH, familial hypercholesterolemia; CAD, coronary artery disease; HTN, hypertension; T2DM, type 2 diabetes mellitus.

The lipids, medical history, and lipid-lowering medication situation in **Table 1** were documented at the time of recruiting.

**Table 2:** Damaging mutations found for the proband using WES.

Chromosome	19p13.2	2p24.1
Position	11227552	21232016
ID	rs1205480064	rs201152495
Gene	*LDLR*	*APOB*
Mutation	(Exon12): c.1723C>T	(Exon26): c.7724A>T
Amino acid change	p.(Leu575Phe)	p.(Lys2575Ile)
Variation type	Missense	Missense
OMIM	606945	107730
Clinvar	Likely pathogenic	VUS
GenomAD_exome	0.000004	0.000116
TOPMED	0.000016	0.000151
ExAC_ALL	–	0.0001
G1000	–	0.000199681
SIFT	D	D
Polyphen2_HDIV	D	B

OMIM, online Mendelian inheritance in man; Clinvar, public archive of relationships among sequence variation and human phenotype; GenomeAD_exome, allele frequency of the mutation of the Genome Aggregation Database; TOPMED, Trans-Omics for Precision Medicine; ExAC_ALL, allele frequency of the mutation in the Exome Aggregation Consortium (ExAC) Browse; G1000, 1000 Genomes Project.

### Metagenomics Analysis Comparing Familial Hypercholesterolemia and Unaffected Hyperlipidemia Subjects in This Family

**Figure 2:**
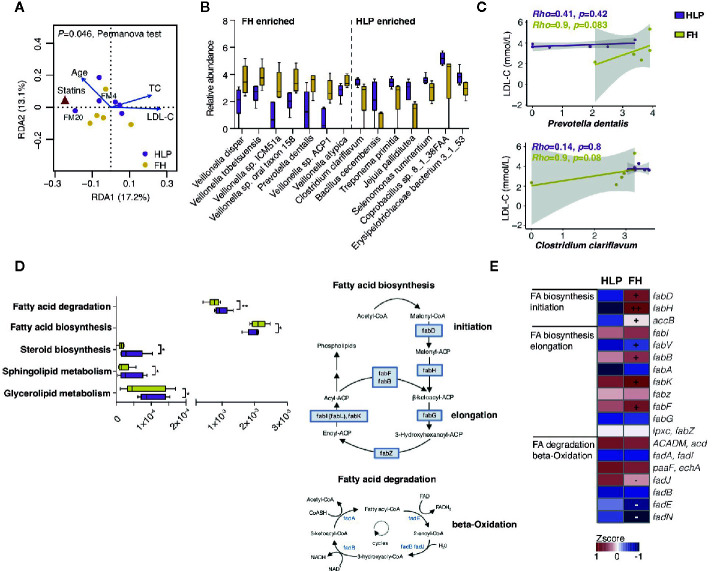
Metagenomics profile of this HeFH family. **(A)** Distance-based redundancy analysis (dbRDA) plot based on clinical parameters fitted to the variation in gut microbiota. **(B)** Relative abundance of significantly differed species between FH and HLP individuals (P < 0.05, Wilcoxon rank sum test). Boxes represent the inter-quartile ranges, and lines inside the boxes denote medians. **(C)** Spearman correlations between bacterial relative abundance and LDL-C levels. **(D)** Alterations in gut microbial lipid functional modules. *P < 0.05; **P < 0.01. **(E)** KO genes significantly changed between FH and HLP individuals are shown in the heat map. Significant changes (elevation and depletion) are denoted as follows: +, elevation with P < 0.05; −, depletion with P < 0.05.

Since several members without mutation of *LDLR* c.1723C>T in this family were also present with dyslipidemia, we then investigated the changes in the gut microbiome in the FH members with mutation of *LDLR* c.1723C>T (n = 5, FM1, FM2, FM4, FM6, and FM11) and unaffected members with hyperlipidemia (HLP, n = 6, FM12, FM17, FM18, FM20, FM21, and FM22) by metagenomics sequencing, to further explore the contribution of microbial communities to cholesterol homeostasis. Redundancy analysis indicated that age, statins therapy, and LDL-C level were the determinant factor for explaining the variations in the bacterial compositions (*P* = 0.046, PerMANOVA test with 999 permutations) (**Figure 2A**). Fourteen bacterial species were observed to be significantly altered (adjusted *P* < 0.05 in Wilcoxon rank sum test and |log2 fold change|> 1). Compared to HLP, the gut microbiota in FH patients harbored increased *Veillonella* sp. and *Prevotella dentalis* (**Figure 2B**). Moreover, we found that *Prevotella dentalis* showed significantly stronger positive correlation with LDL-C level only in FH individuals (**Figure 2C**). *Clostridium clariflavum* also had trendy positive correlation with LDL-C level in FH individuals (**Figure 2C**). FH- depleted microbial function included degradation of module of fatty acid, whereas increase of fatty acid initiation and elongation biosynthesis (**Figures 2D, E**). The bacterial fatty acid synthesis pathway has significant potential as a target for novel drugs and β-hydroxyacyl-ACP of the proper length is used for lipopolysaccharide (LPS) synthesis in Gram-negative bacteria (Heath and Rock, 2004).

## Discussion

Here, we unmasked the genetic cause of a young male patient with early-onset MI through detailed clinical assessment and diagnosed other nine family members with HeFH in the extended family through WES analysis. The *LDLR* c.1723C>T variant was classified as likely pathogenic based on family co-segregation analyses. It was once detected in a young Chinese patient with FH in 2016 (Jiang et al., 2016).This mutation is in the exon 12 which is within the EGF precursor homology domain (i.e. exon 7–14), and is involved in the dissociation of the receptor and the lipoprotein in the endocytosis machinery (Zhang et al., 2007). Our study revealed that genomic test along with family screening, is confirmatory and clinically relevant, especially for individuals with borderline-high LDL-C levels.

In the current Chinese family cohort, the specific genetic relatedness help dissect the gut microbial variation between FH and hypercholesterolemia. We discovered that microbiota was affected by statins therapy in accordance with other research that higher gut biodiversity was associated with statin sensitive response (Sun et al., 2018). Statins therapy has been reported as a key covariate of modulating the prevalence of *Prevotella* sp. (Vieira-Silva et al., 2020). Similarly, the *Veillonella *sp. enriched in FH individuals are often present in the oral cavity and is identified to be abundant in hyperlipidemia cohorts (Mashima et al., 2016; Liu et al., 2019). Recent study indicated that microbiota is implicated in human genetic adaptations and host mechanisms can replace or recruit beneficial microbiota functions during local adaptation (Suzuki and Ley, 2020). We speculated that host gene-microbe interactions and lipids metabolic disorders caused by LDLR mutations may disturb bacteria community homeostasis in FH individuals, resulting in positive correlation between specific bacteria and LDL-C level. *Prevotella* sp. is regarded as potential pathogens for CVD (Liu et al., 2019), and Clostridiaceae was found associated with lipid profile in previously large cohort (Fu et al., 2015). In addition, bacterial fatty acid synthesis is a vital facet of bacterial physiology, whereas the intermediate metabolite could catalyze the initial step in the biosynthesis of LPS (Li et al., 2003; Yao and Rock, 2015). The gut microbiota in FH individuals may exhibit stronger function of pro-inflammatory potential which facilitate lipometabolic disturbance. However, more FH family trios are necessary in order to decipher the microbial predisposition and the mechanistic participation of genotype-microbiota interaction remain to be further biologically elucidated.

Overall, we showed a potential contribution of dysbiotic gut microbiota to the cholesterol disorder associated with genetically predisposed hyperlipidemia, as with simple hyperlipidemia although CAD risk factors and medications might imprint their effects microbiota. Whole exome sequencing in combination with clinical criteria facilitate early diagnosis of FH which can lead to preventive treatment that reduce cardiovascular risk. Our results characterize complex genetic and microbial variation in the regulation of cholesterol biological processes. This study provides conceptual insights that lay important groundwork for future applications in rare diseases, which will have to take into account both the genome and metagenome.

## Data Availability Statement

The datasets presented in this study can be found in online repositories. The metagenomics raw sequence data have been deposited in the Genome Sequence Archive under accession number CRA002675 at https://bigd.big.ac.cn/gsa.

## Ethics Statement

The studies involving human participants were reviewed and approved by the Peking Union Medical College Hospital. Written informed consent was obtained from the individuals for the publication of any potentially identifiable data included in this article.

## Author Contributions

HL and YJ conceived and designed the analysis. RT and SF collected the samples and data. HL and CZ performed the analysis. HL, CZ, and SZ wrote the manuscript. YFW and YYW revised the manuscript. All authors contributed to the article and approved the submitted version.

## Funding

This work was supported by the National Natural Science Foundation of China (81670329), Center for Rare Diseases Research, Chinese Academy of Medical Sciences, Beijing, China (Grant No. 2016ZX310174–4), CAMS Innovation Fund for Medical Sciences (CIFMS) (No. 2016-I2M-1-011 and No. 2017-I2M-2-001).

## Conflict of Interest

The authors declare that the research was conducted in the absence of any commercial or financial relationships that could be construed as a potential conflict of interest.
